# Phenology and Diversity of Weeds in the Agriculture and Horticulture Cropping Systems of Indian Western Himalayas: Understanding Implications for Agro-Ecosystems

**DOI:** 10.3390/plants12061222

**Published:** 2023-03-08

**Authors:** Shiekh Marifatul Haq, Fayaz A. Lone, Manoj Kumar, Eduardo Soares Calixto, Muhammad Waheed, Ryan Casini, Eman A. Mahmoud, Hosam O. Elansary

**Affiliations:** 1 Department of Ethnobotany, Institute of Botany, Ilia State University, Tbilisi 0162, Georgia; 2 Department of Botany, Government Degree College (Women), Kupwara 193222, India; 3 GIS Centre, Forest Research Institute (FRI), PO New Forest, Dehradun 248006, India; 4 Institute of Food and Agricultural Sciences, University of Florida, Gainesville, FL 32962, USA; 5 Department of Botany, University of Okara, Okara 56300, Pakistan; 6 School of Public Health, University of California, Berkeley, 2121 Berkeley Way, Berkeley, CA 94704, USA; 7 Department of Food Industries, Faculty of Agriculture, Damietta University, Damietta 34511, Egypt; 8 Department of Plant Production, College of Food & Agriculture Sciences, King Saud University, P.O. Box 2460, Riyadh 11451, Saudi Arabia

**Keywords:** agriculture, weed survey, weed management, beta diversity, indicator species analysis

## Abstract

Weeds are a major threat to agriculture and horticulture cropping systems that reduce yield. Weeds have a better ability to compete for resources compared to the main crops of various agro-ecosystems and act as a major impediment in reducing overall yield. They often act as energy drains in the managed agroecosystems. We studied weed infestation for five different agro-ecosystems in the part of Indian Western Himalayas represented by paddy, maize, mustard, apple and vegetable orchards. Systematic random sampling was done to record flowering phenology and diversity of weeds during the assessment period 2015–2020. We recorded 59 weed species, taxonomically distributed among 50 genera in 24 families. The Asteraceae family has the most species (15% species), followed by Poaceae (14% species), and Brassicaceae (12% species). The Therophytes were the dominant life form followed by Hemicryptophytes. The majority of the weeds were shown to be at their most blooming in the summer (predominantly from June to July). The Shannon index based diversity of weeds ranged from 2.307–3.325 for the different agro-ecosystems. The highest number of weeds was in the horticulture systems (apple > vegetable) followed by agriculture fields (maize > paddy > mustard). Agriculture and horticulture cropping systems were distinguished using indicator species analysis, which was supported by high and significant indicator values for a number of species. *Persicaria hydropiper*, *Cynodon dactylon*, *Poa annua*, *Stellaria media*, and *Rorippa palustris* had the highest indicator value in agriculture cropping systems, while *Trifolium repens*, *Phleum pratense*, and *Trifolium pratense* had the highest indicator value in horticulture cropping systems. We found that eleven weed species were unique to apple gardens followed by nine in maize, four in vegetables, two in mustard and one in paddy fields. Spatial turnover (βsim) and nestedness-resultant components (βsne) of species dissimilarity revealed dissimilarity lower than 50% among the five cropping systems. The study is expected to assist in formulating an appropriate management strategy for the control of weed infestation in the study region.

## 1. Introduction

The vegetated land can broadly be categorized into forested and agricultural landscapes. In vegetated lands, weed invasion has been referred to as one of the prominent threats after climate change [1,2,3,4]. Weeds are a group of specialized plants that have evolved along temporal and spatial scales because of their large seed production, aggressive reproduction, high regenerative capacity, and large phenotypic plasticity [5]. The weed plants are considered unwanted and undesirable at a particular site [6,7]. Among the various biological stresses, weeds are known as one of the most detrimental to crop production [8,9]. In addition, these weeds provide shelter to numerous pests of crop plants and, thus, indirectly become a cause of many crop diseases [10]. Weeds are notorious for decreasing crop yield and are found to be economically more detrimental than bacteria, fungi, insects or other crop pests in many situations. For instance, it has been estimated that weeds result in about USD 11 billion of economic losses every year in India [11].

Weeds often invade human-controlled settings, such as agricultural fields, orchards, parks, and lawns. The presence of weeds in agricultural settings is well-known from the beginning of civilization; however, their role in agriculture and horticulture is recognized only in recent decades [12]. The broad ecological amplitude of weeds facilitates them to invade a broad range of habitats and ecological niches. The presence of weeds in the agricultural and horticulture fields compete with the native plants to affect yield [10]. Although horticulture is considered one of the subdivisions of agriculture, they differ in management practices and the kind of plants grown. Thus, it might be possible that similar land under a different regime of management for growing different crops may influence selective weed infestation. This provides an opportunity to investigate the weed invasion in these two different land use classes. At the same time, the information on the phenological events of weed is important for formulating effective control and management [13,14].

The documentation of weeds in different crop fields is important for the management and control of weeds [15,16]. The identification and documentation of weed invasion are important for formulating various strategies for managing the weeds [17]. Management decisions for agriculture and horticulture fields are dependent upon the phenological information of crops for improving yield [13] while the phenological calendar of weeds is important for formulating their effective eradication strategy. In the study landscape, most of the weeds are invasive and not native to the place. There is a need to understand the type, pattern, and impacts of weeds and invasive species for improving the targeted yields of the selected cropping system. However, there is a wide knowledge gap in invasive species research in developing countries and it acts as a decisive impediment to managing invasion. Developing countries lag far behind developed countries in invasive species research [18]. Specifically, Asian countries are represented poorly in the scientific literature on invasive species. Such a lag and data deficit must not be considered an indicator of low invasion risk or low intensity of invasion in developing countries. Rather, this indicates less effort being made to explore the invasive species, less documentation, and insufficient action on data availability.

For supplementing the existing knowledge base on invasive weeds, we present here a comprehensive assessment to achieve the objectives of (i) documenting various weeds in agriculture (paddy, maize, and mustard) and horticulture (apple and vegetable) systems of the study region, (ii) identification as native vs. exotic, (iii) diversity assessment of weed under different cropping systems, and (iv) understanding flowering phenology of weeds. We also attempt to address research queries on crop specificity of weeds to a given cropping system and to assess whether a specific cropping system has more diverse weeds than another. The findings will supplement existing knowledge on invasive weeds amid a dearth of knowledge for the study region. At the same time, a similar approach can be adopted for collecting vital information on weeds for other study regions.

## 2. Results

The study recorded 59 weed species, taxonomically distributed among 50 genera in 24 families (Table 1). The perennial weeds were 28 in number constituting 48% of the total encountered weeds in all of the cropping systems. The other life span categories were annual (39%), annual–biennial (7%), and annual–biennial–perennials (3%) having 23, 4, and 2 weed species, respectively (Table 1). The distribution of species among 24 families is lopsided, with 4 families accounting for half of the species and 20 families for the other half. 15 families were represented by the presence of just single species. The Asteraceae was the dominant family with 9 species (15%) followed by Poaceae with 8 species (14%), Brassicaceae with 7 species (12%), and Plantaginaceae with 5 species (8%). The rest of the species was represented by Fabaceae, Lamiaceae, Polygonaceae, Ranunculaceae, Amaranthaceae, and other families (Table 1). The monotypic families are Apiaceae, Boraginaceae, Asparagaceae, Caryophyllaceae, Chenopodiaceae, Cyperaceae, Euphorbiaceae, Fumariaceae, Geraniaceae, Rosaceae. Further enumeration is represented in Table 1.

### 2.1. Functional Traits Including Flowering Phenology

**Figure 1:**
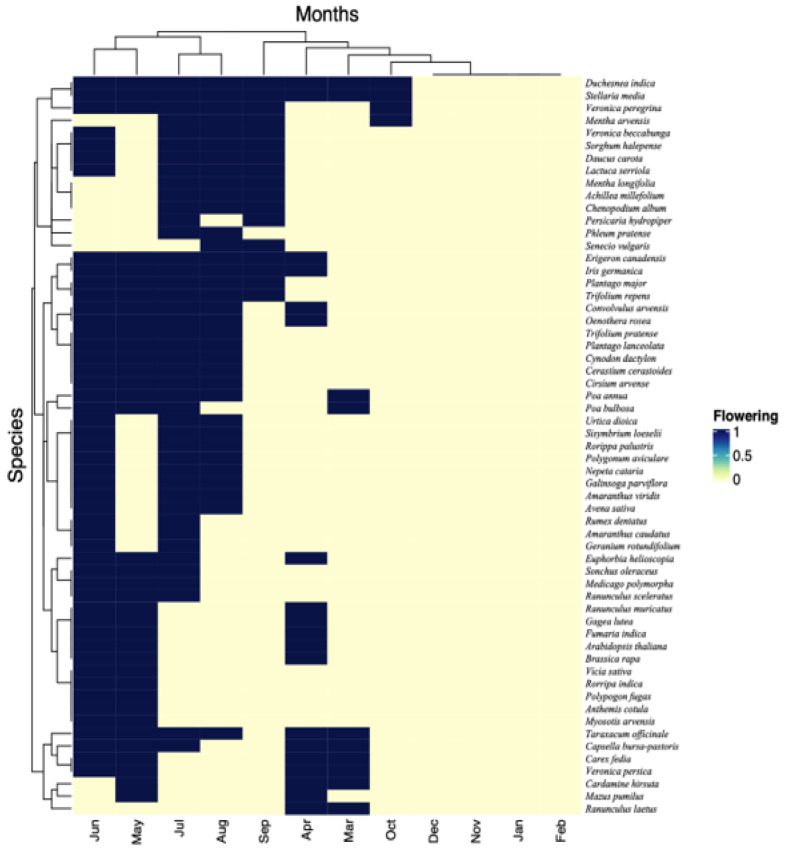
Two-way cluster analysis based on Sorenson’s similarity index and flowering phenology of weeds.

The analysis of floristic distribution using Raunkiaer’s life form revealed that the therophytes with 35 species forming 59% of the plant community were the dominant life form in the study region, followed by hemicryptophytes with 19 (32%), and geophytes with 5 (9%) (Table 1). The phytogeographical analysis revealed that the maximum weed species (34 in number, 58%) recorded is native, while many (25 in number, 42%) are alien (Table 1). The phenological spectrum of weed flora was presented mainly by the flowering period of each species. The present study’s weed flora displayed a wide range of blooming phenology (Table 1, Figure 1). Different species flower through various seasons. We observed that most of the weeds (77%) (e.g., *Achillea millefolium*, *Amaranthus viridis*, *Avena sativa*, *Daucus carota*, *Galinsoga parviflora*, *Geranium rotundifolium*, *Nepeta cataria*, *Polygonum aviculare*, *Achillea millefolium*, *Lactuca serriola*, *Persicaria hydropiper* and *Phleum pratense*) flowered from May to August (Table 1, Figure 1). A few weeds showed flowers in other months of the year, such as *Brassica rapa*, *Capsella bursa-pastoris*, *Cardamine hirsuta*, *Ranunculus arvensis*, *Mazus pumilus*, *Rumex hastatus*, *Viola odorata*, *Tussilago farfara* and *Sigesbeckia orientalis* which bloomed between September and April. This observed variance in the phenological response of blooming across different weeds was ascribed to seasonal temperature variations. The majority of weeds exhibited their peak blooming in the summer, primarily in June and July. The blossoming season typically began at the beginning of spring and lasted until the end of summer (Table 1, Figure 1). The clustering of weeds based on flowering phenology is presented in Figure 1, where weeds grouped in one limb are more similar in flowering timings and show proximity to each other.

### 2.2. Weed Distribution and Their Diversity in Agriculture and Horticulture Systems

The horticulture management interventions in the apple and vegetable orchards were found to have more weeds compared to the agriculture systems. Among agriculture systems, maize has the highest number of weeds followed by mustard and paddy. The apple garden had significantly higher weed diversity (3.325) than the mustard (2.307). The value of weed dominance based on the Simpson index ranges from 0.835 to 0.955 (Table 2). The other indices of weed diversity in five cropping systems are presented in Table 2.

### 2.3. Indicator Species Analysis

**Figure 2:**
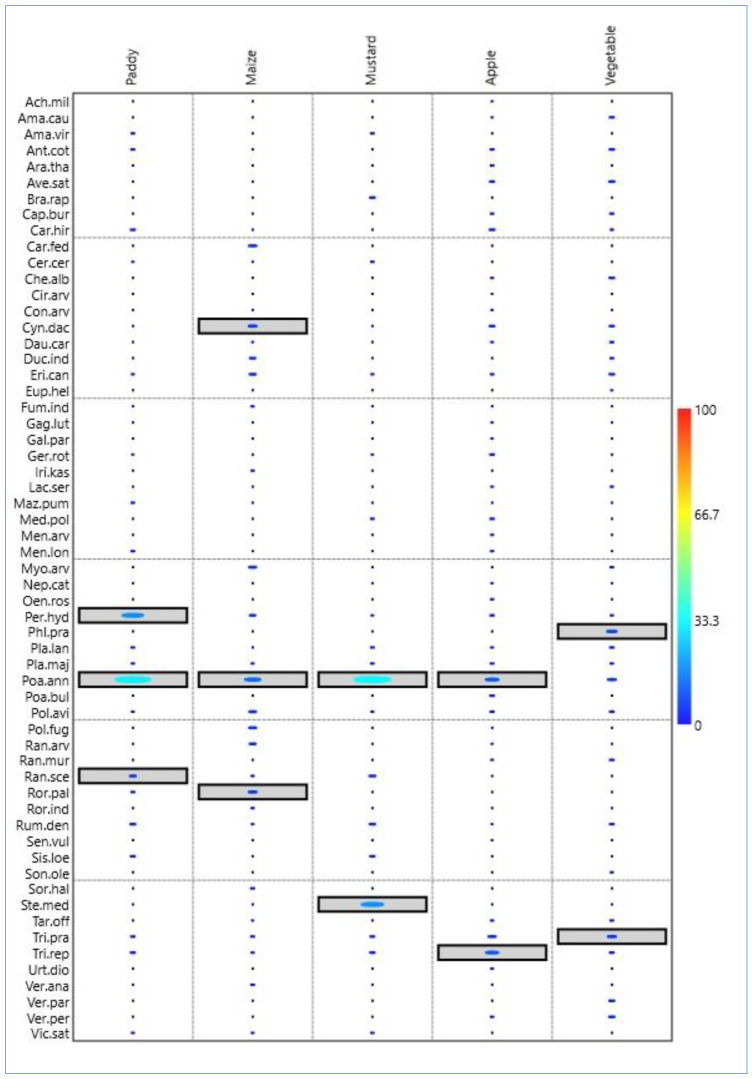
Indicator species analysis diagram showing the most significant indicator species in the agriculture and horticulture systems (paddy, maize, mustard, apple, vegetable). Horizonal lengths show contribution of individual species as indicator values based on combining values for relative abundance, relative density and relative frequency, and *p*-values are shown with bar colour (0 to 100) representing Monte Carlo test of significance of the observed maximum indicator value for each species. The highest indicator values are shown within rectangles. The importance value index for indicator species were as *Poa annua* (292), followed by *Persicaria hydropiper* (91), *Trifolium repens* (73), *Trifolium pratense* (69), *Stellaria media* (59), *Cynodon dactylon* (46), *Ranunculus sceleratus* (38), *Rorippa palustris* (30), and *Phleum pratense* (25). The bar in the figure shows the indicator species with a significant *p*-value. The bigger the dot, the more significant the *p*-value of the species.

The indicator species analysis showed the separation between the paddy, maize, and mustard from apple garden and vegetable fields, as evidenced by high and substantial indicator values for several species. In the paddy field, *Persicaria hydropiper* and *Poa annua* had the highest indication value, while *Cynodon dactylon*, *Poa annua*, and *Rorippa palustris* had the highest indicator value for maize fields. In mustard fields, the indicator species with significant p-value were *Stellaria media* and *Poa annua*. *Trifolium repens* and *Poa annua* had the highest indicator value in the apple garden, while in vegetable fields, the *Phleum pratense* and *Trifolium pratense* had the highest indicator value. *Poa annua* was the indicator species in all four types of agriculture fields excluding vegetable fields (Figure 2).

**Figure 3:**
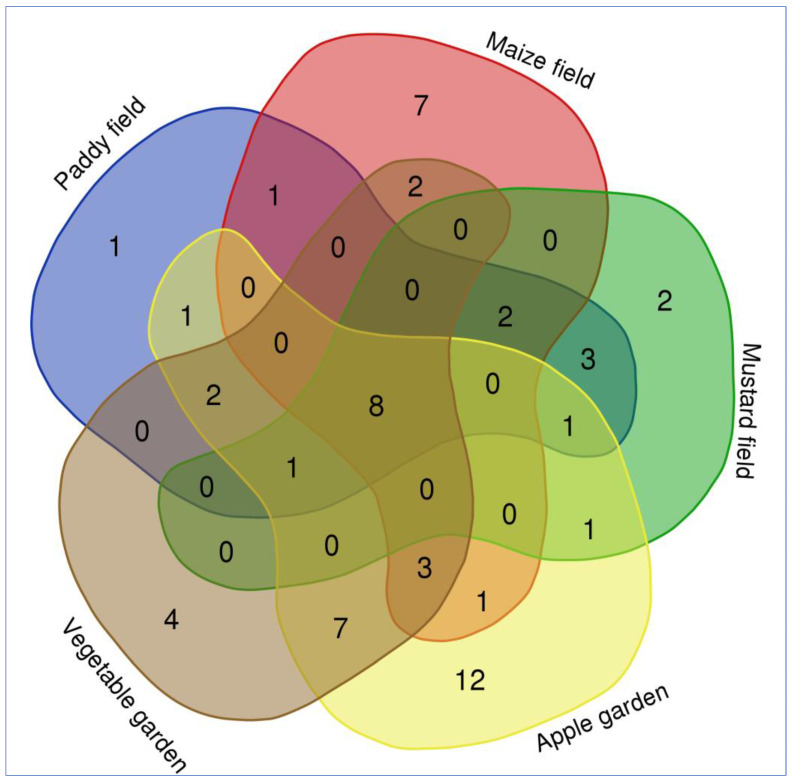
Venn diagram showing the distribution of the number of weed species occurring in different cropping systems of Kupwara district, Jammu and Kashmir, India.

**Figure 4:**
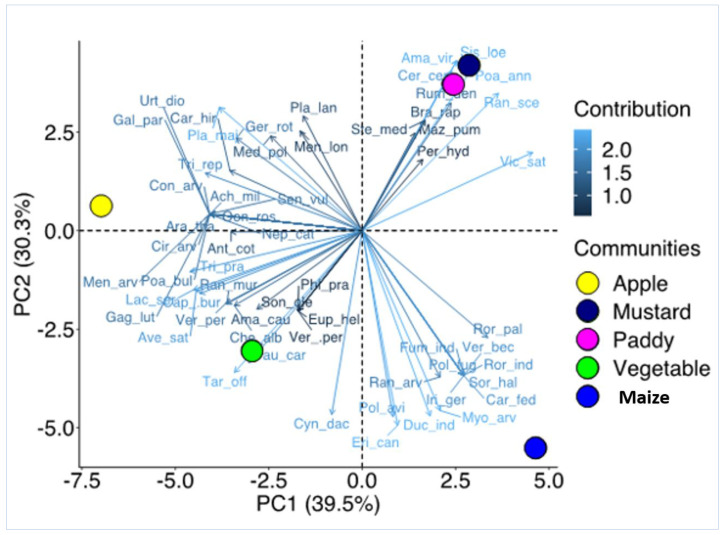
Principle Component Analysis (PCA) illustrating the relationship of weed species and the five cropping systems evaluated for the Kupwara district, Jammu and Kashmir, India. The contribution scale shows how much each weed species contributes to the PCA. (Full name of the species can be referred to in Table 1).

It was observed that eleven weed species were unique to the apple garden followed by nine species in the maize field, four species in the vegetable garden, two species in the mustard field, and one species in the paddy field (Figure 3). However, eight species were common in all habitat types that included *Erigeron canadensis* L., *Persicaria hydropiper* Delarbre, *Plantago major* L., *Poa annua* L., *Polygonum aviculare* L., *Rumex dentatus* L., *Trifolium pratense* L., *Trifolium repens* L. Similarly, nine species were common between apple and vegetable orchards. Three weed species were common between apple, vegetable, and maize cropping systems. One weed species was common between a maize field and a vegetable orchard. The Venn diagram depicted in Figure 4 shows the number of weed species unique to a specific cropping system and common among different cropping systems. PCA analysis showed four distinct groups based on the composition and IVI of the weed species. Apple, vegetables, and maize were distinctly separated from each other, while mustard and paddy had a similar composition of weeds (Figure 4).

The species *Achillea millefolium*, *Arabidopsis thaliana*, *Cirsium arvense*, *Convolvulus arvensis*, *Gagea elegans*, *Oenothera rosea*, *Poa bulbosa*, *Nepeta cataria*, *Senecio vulgaris*, *Medicago polymorpha*, *Urticadioica* are unique to apple growing fields and species *Carexfedia*, *Fumaria indica*, *Sorghum halepense*, *Rorippaindica*, *Iris germanica*, *Polypogon fugax*, *Veronica anagallis-aquatica*, *Myosotis arvensis* were unique to vegetable fields. Weed plants such as *Carex fedia*, *Fumaria indica*, *Sorghum halepense*, *Rorippaindica*, *Iris germanica*, *Polypogon fugax*, *Veronica anagallis-aquatica*, *Myosotis arvensis* are specific to the maize field. The species linked to paddy and mustard fields are *Lactuca serriola*, *Mentha arvensis*, *Amaranthus caudatus*, *Avena sativa*, *Capsella bursa-pastoris*, *Ranunculus muricatus*, *Chenopodium album*, *Veronica persica*, *Galinsoga parviflora* (Figure 4).

**Figure 5:**
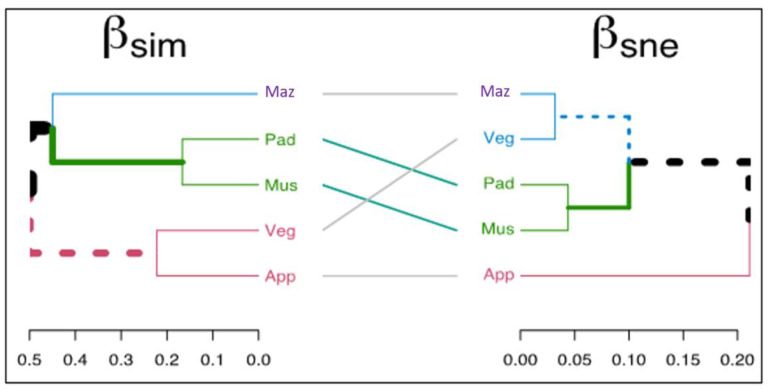
Dissimilarity cluster based on spatial turnover (βsim) and nestedness-resultant components (βsne) of beta diversity components of weeds in five different cropping systems of Kupwara district, Jammu and Kashmir, India. (App—Apple, Veg—Vegetable, Mus—Mustard, Pad—Paddy, Maz—Maize).

Spatial turnover (βsim) and nestedness-resultant components (βsne) of species dissimilarity revealed dissimilarity lower than 50% among the five crops (Figure 5). In the βsim cluster, we observed three distinct clusters; one made up of apple and vegetable, another of paddy and mustard, and the last one only of maize. Maize showed a difference of 44–50% in weed composition when compared to the other four crops. Apple crops showed a difference in weed composition of 35% and 38% when compared to paddy and mustard, respectively. In addition, vegetable crops had 45% and 50% different weed species when compared to paddy and mustard, respectively. On the other hand, the highest value in the βsne cluster was 21%, showing that the number of species between crops is similar, differing by less than 21%. This highest value was found between apple and mustard. Moreover, the number of weed species in apple crops was 19% different from paddy. The other relationships between crops regarding βsne had less than a 12% difference in weed species number.

## 3. Discussion

The first step in dealing with the problem of weeds is their identification and documentation not just for a specific location or region but also for the specific cropping systems. The current study would help in formulating management strategies in dealing with weeds. We could find only a limited such studies for the part of IWH while there is a lack of information on weed for most of the regions of India. The findings will help in an improved understanding of weed infestation under the different agroecosystems. As the foremost and important step in weed management is the proper identification of different weeds, their phenological attributes, and habitat preference; the present study provides essential insight for effective weed management.

The presence of weeds in agricultural settings drains nutrients and moisture from the soil and prevents sunlight to reach the plants [19]. As a result, it decreases the yield of crops significantly [20]. Thus, it is imperative to understand the distribution pattern and diversity of weeds in different cropping systems for their effective management to sustain crop yield [10]. The number of species found in the present study is more than what other researchers in other Himalayan locations have discovered. A total of 35 weeds from 33 genera and 18 families were recorded by Khan et al. [21] from the Ochawala valley in the Pakistani district of Charsadda. According to Haq et al. [22], the Pakistani district of Nowshera’s onion crop had a total of 21 species. In the Pakistani Himalayas’ Mohmand Agency, Ali et al. [23] discovered 63 weed species. We could not find such studies for our study region, i.e., the Kupwara district of Jammu and Kashmir, India. A higher number of weed species was reported from the families belonging to Asteraceae, Poaceae, and Brassicaceae. The Asteraceae family shows habitat diversity because of its wide ecological amplitude. Khan et al. [21] reported Poaceae as the dominant weed family from Pakistan Himalayas. Several researchers reported Asteraceae as the dominant family of weeds from other regions [24,25,26]. Interestingly, other Himalayan regions were also dominantly occupied with the above-mentioned families as reported in previous studies [21,27]. The current analysis reveals an uneven distribution of species among families, and 9 of those families were monotypic. These results are fairly equivalent to prior reported values from other western Himalayan locations [21,24,28].

The comparative percentage of dissimilar life form to an existing flora in a specific region or spot is called its biological spectrum [29]. The biological spectrum reveals how plants have changed to cope with their micro- and macro-climates, and it contains significant physiognomic traits that are frequently used in vegetation research [30,31]. The most common group of biological forms were therophytes, followed by hemicryptophytes and geophytes. Therophytes, which are the dominating life form in the studied region, is a sign of significant biotic disturbances on the habitat caused by activities such as grazing, farming, road building, etc. This life form is typically linked to adverse dry environmental conditions [32,33,34,35]. Our findings agree with the previous studies [36,37,38]. Therophytes dominating in such disturbed habitat zones increase the number of species through the introduction of alien annual weedy forbs such as *Anthemis cotula*, *Amaranthus caudatus*, *Galinsoga parviflora*, etc. [33,39]. The plausible reason for the predominance of *hemicryptophytes* is due to the available open space and high rate of reproduction [40]. Due to their deep perennial portions, the geophytes only emerge during a brief spring and stay dormant during adverse seasons [41].

The paddy, maize, and mustard were separated from apple orchards and vegetable fields according to the indicator species analysis, which was supported by high and significant indicator values for a number of species. Interesting, each Venn diagram created for each type of habitat across the cropping systems showed a similar pattern (Figure 3). The results from the Venn diagram depicted that eleven species were unique to the apple garden followed by nine species in the maize field, four species in the vegetable garden, two species in the mustard field and one species in the paddy field. The consequence of differential habitat selection results from evolutionary adjustment of species to environmental variables [42]. Species establish under the prolonged prevailing circumstances to function better in a cropping habitat relative to the other habitats [43].

## 4. Materials and Methods

The study involved field-based surveys for five consecutive years (2015–2020) to collect information on weed listing, diversity assessment under different cropping systems, and flowering phenology. The standard taxonomic procedure was followed for the collection of plant specimens. The specimens were identified using relevant taxonomic literature and were further authenticated by matching the plant specimens kept in the herbarium of the Centre for Biodiversity and Taxonomy, University of Kashmir. The herbarium of the center has been recognized by the International Bureau for Plant Taxonomy and Nomenclature, New York with acronym KASH (http://taxonomy.uok.edu.in/Main/AboutUs.aspx/ accessed on 8 May 2022). Details on sampling and analysis are discussed in this section ahead.

### 4.1. Study Area

**Figure 6:**
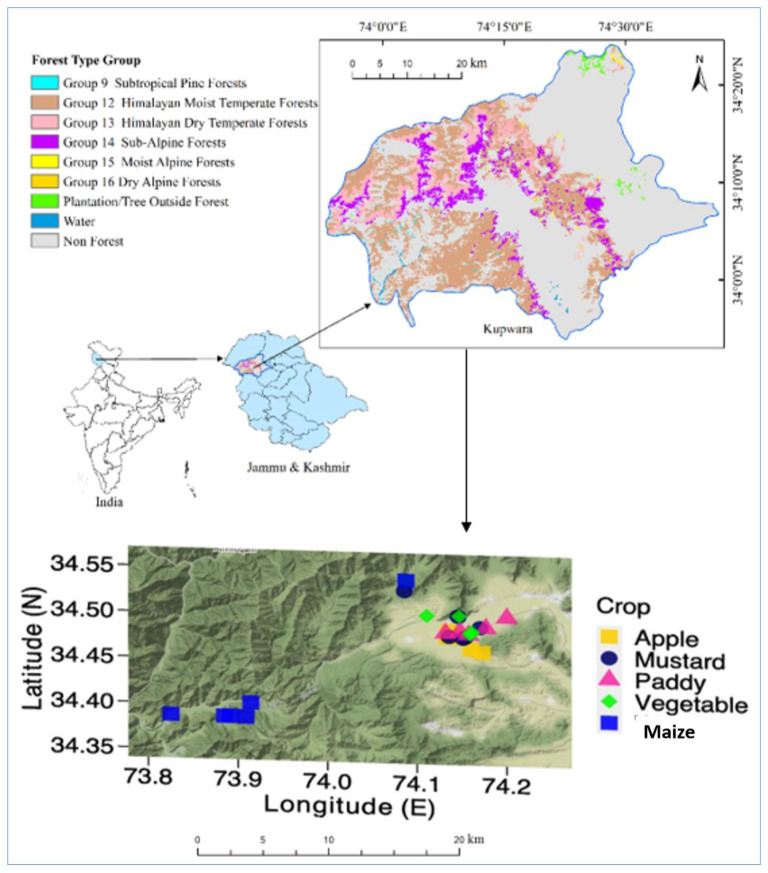
Map of the study area showing the location with dominant forest types in upper panel and lower panel showing sampling sites in the Kupwara district, Jammu and Kashmir state, India. The locations of apple, mustard, paddy, vegetable, and maize sites are indicative of sampling sites that included winter, summer and rainy season sampling and they do not represent the number of samples.

The study was conducted for the Kupwara district of Jammu and Kashmir state, located in the Indian Western Himalayas (IWH). The IWH represents one of the most sensitive ecosystems (http://www.knowledgeportal-nmshe.in/ accessed on 8 May 2022) with very rich biodiversity where many species are rare and endemic [3]. At the same time, the region faces the threat of weed invasion making it vulnerable to ill effects of invasion while the other factors making the region vulnerable include proximity to streams, slopes, and elevations [44]. District Kupwara is the northernmost geographical part of the Kashmir Himalaya in the IWH. Its geographical area is 2379 km^2^, situated at a height of around 1616 m amsl. The region extends between the latitudes of 34.17–34.21 E and the longitudes of 73.10–73.16N (Figure 6). It is the backward frontier of the Kashmir Himalayas, with a line of control abutting it to the northwest. The river Kishenganga, originates in the Himalayas and flows from east to west, through the outer areas of Kupwara. The region harbours a large number of weeds because of a broad range of physical land features having large climatic variations across the region. In addition, this part of Himalaya has diverse cropping pattern with a large variety of cultivated crops where few weeds are recognised as crop specific weed [45]. The dominant vegetation of the area is represented by Himalayan dry temperate forests, Himalayan moist temperate forests and Sub-Alpine forests [46]. Scrub forest vegetation intersperse the different forest types at higher altitudes. Patches of grassland meadows are also quite common here (Figure 6).

### 4.2. Sampling Design and Analysis

**Figure 7:**
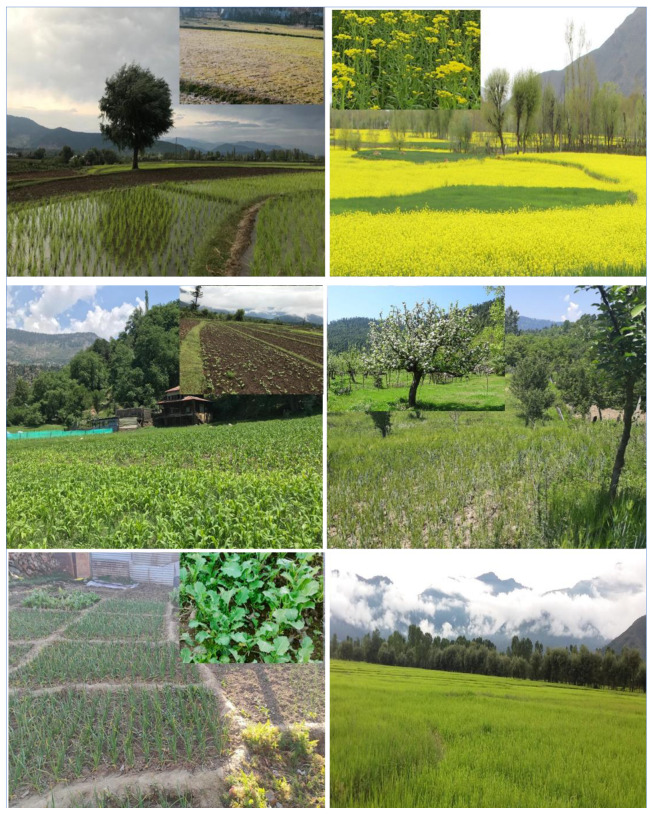
The photograph depicts the studied agriculture and horticulture systems in the study area, 1. (**Top left**): paddy field in winter and summer season, 2. (**Top right**): mustard field in rainy and summer season, 3. (**Middle left**): maize field in summer and rainy season, 4. (**Middle right**): apple garden in rainy and summer season, 5. (**Bottom left**): vegetable garden in winter and summer season, 6. (**Bottom right**): paddy field in rainy season.

Preliminary field surveys were carried out to obtain an understanding about the nature of the terrain, vegetation, distribution and accessibility in the study area. After that, five cropping types in two distinct systems of agriculture and horticulture were identified for the assessment. The agriculture system constituted of paddy, maize and mustard cultivation while the horticulture system consisted of apple and vegetable gardens. Systematic random vegetation sampling was carried out to record floral diversity in the different habitats during the year 2015–2020. In total, 250 quadrates of size 5 m × 5 m were laid down for sampling. This constituted 50 plots in each of the five selected cropping systems. The sampling was done to ensure field enumeration for all of the three distinct seasons (i.e., winter, summer, rainy) as few of the weed species were not perennial and their occurrence was reported only during a specific season **(Figure 7).** The selection of plot size was carried out by drawing species vs. area curve, where we found that the number of weed species presence counted was usually saturated at the selected size of 5 m × 5 m size. Species listing of weeds was simply taken as the count of the total number of different weeds occurring in all of the sampled study quadrates for a selected cropping system. The dominating weed species present in a particular agricultural system were identified using the Importance Value Index (IVI) of weeds (Supplementary Materials). The IVI was calculated by adding the relative frequency, relative abundance, and relative density of each weed species in a given cropping system [47]. Field notebooks were used to record in-depth field observations on ecological characteristics for each species, such as blooming phenology, habit, and Raunkiaer’s life form [48,49]. The native phytogeographical distribution of the plant species gathered from the study region was obtained using secondary sources such as floras, manuals, and recently published research papers [45] and specialized internet web pages of Germplasm Resource Information Network (GREEN) https://www.ars-grin.gov/ accessed on 8 May 2022 and www.efloras.org/ accessed on 8 May 2022. Based on data sources that were accessible, plant species were divided into native and alien species. The diversity indices i.e., Shannon–Wiener index [50], Simpson diversity index [51], and Evenness index [52] were calculated using the standard formula.

The Venn diagram evaluates the unique and common species among cropping systems by using Bioinformatics & Evolutionary Genomics tool (available at http://bioinformatics.psb.ugent.be/webtools/Venn/ accessed on 5 June 2022). Principal Component Analysis (PCA) was done to visualize the weed associations between crops using the package “vegan” in the software R 4.0.0 [53,54]. In order to compare the β-diversity of weeds at the habitat and landscape levels, we also calculated β-diversity, which was produced as a ratio of the regional and local diversity. We used the spatial turnover (Simpson pairwise dissimilarity) and nestedness-resultant components (nestedness-fraction of Sorensen pairwise dissimilarity) of β-diversity analysis applying “Sorensen” as family of dissimilarity index while the Dissimilarity analysis was conducted in the package “betapart” [55]. In addition, indicator species analysis was used to identify the key weed species for each habitat type using PAST software [56]. Indicator values were computed according to Dufrêne and Legendre [57], and the statistical significance of the maximum indicator value was determined using the Monte Carlo Test of significance [58].

## 5. Conclusions

This study provides a comprehensive understanding of the distribution and diversity of weeds under the various management interventions that distinguish cropping systems from agriculture and horticulture systems. The findings are expected to assist farm managers to develop effective management plans to control and eradicate weeds infestation in the paddy, maize, mustard, apple, and vegetable agro-ecosystems of the IWH region. Such studies provide essential information to manage weeds where lack of sufficient data on weeds is one of the major constraints for formulating an effective weed management plan. The flowering phenology of weeds will help to establish an effective time for the application of herbicides or to implement manual weeding operations (i.e., removal of weeds) in the field, as the most appropriate weeding time is before the flowering of weeds. The dominant weeds and their clustered flowering phenological timings in a year for each of the dominant cropping systems will further help to identify an ideal time in a year for implementing weeding operations to achieve optimum results of weed eradication.

## Figures and Tables

**Table 1:** Description of weeds in the part of Indian Western Himalayas (IWH), Kupwara district, Jammu & Kashmir, represented by their scientific name, family, dominance in different cropping systems shown by the Important Value Index (IVI), origin (native/exotic), flowering phenology and Raunkiaer life form. (Life span: A = annual, B = biennial, P = perennial, A-B = annual-biennial, A-B-P = annual-biennial-perennials. Dominant weeds in either of the cropping systems have been highlighted in bold along with their top three IVI values).

Scientific Name	Family	Life Span	Agriculture			Horticulture		Origin	Flowering	Raunkiaer
			Paddy	Maize	Mustard	Apple	Vegetable		Phenology	Lifeform
***Achillea millefolium* Linn.**	Asteraceae	P	0	0	0	2.74	0	Native	July–Sept.	Hemicryptophyte
***Amaranthus caudatus* L.**	Amaranthaceae	P	0	0	0	2.74	11.20	Exotic	June–Aug.	Therophyte
***Amaranthus viridis* L.**	Amaranthaceae	A	7.45	0	7.85	0	0	Exotic	June–Aug.	Therophyte
***Anthemis cotula* L.**	Asteraceae	A	8.35	0	0	8.32	12.13	Exotic	May–June	Therophyte
***Arabidopsis thaliana* (L.) Heynh.**	Brassicaceae	A	0	0	0	7.23	0	Exotic	April–June	Therophyte
***Avena sativa* L.**	Poaceae	A	0	0	0	11.13	14.24	Exotic	June–Aug.	Therophyte
***Brassica rapa* L.**	Brassicaceae	A	0	0	13.26	0	0	Exotic	April–June	Hemicryptophyte
***Capsella bursa-pastoris* (L.) Medik.**	Brassicaceae	A	0	0	0	6.78	9.34	Native	March–July	Therophyte
***Cardamine hirsuta* L.**	Brassicaceae	B	11.01	0	0	13.02	6.05	Native	March–May	Therophyte
***Carex fedia* Nees**	Cyperaceae	P	0	20.63	0	0	0	Native	March–June	Hemicryptophyte
***Cerastium cerastoides* (L.)**	Caryophyllaceae	A	3.76	0	7.19	0	0	Native	May–Aug.	Therophyte
***Chenopodium album* L.**	Chenopodiaceae	A	0	0	0	5.05	12.61	Exotic	July–Sept.	Therophyte
***Cirsium arvense* (L.) Scop.**	Asteraceae	P	0	0	0	2.08	0	Exotic	May–Aug.	Hemicryptophyte
***Convolvulus arvensis* L.**	Convolvulaceae	P	0	0	0	3.37	0	Exotic	April–Aug.	Therophyte
***Cynodon dactylon* (L.) Pers.**	Poaceae	P	0	21.21	0	13.32	11.47	Native	May–Aug.	Hemicryptophyte
***Daucus carota* L.**	Apiaceae	B	0	2.97	0	4.18	8.41	Exotic	June–Sept.	Hemicryptophyte
***Duchesnea indica* (Andrews) Teschem.**	Rosaceae	P	0	14.51	0	0	7.41	Native	March–Oct.	Hemicryptophyte
***Erigeron canadensis* L.**	Asteraceae	A-B	5.17	16.73	5.62	7.28	12.61	Exotic	April–Sept.	Therophyte
***Euphorbia helioscopia* L.**	Euphorbiaceae	A	0	0	0	0	3.29	Exotic	April–July	Therophyte
***Fumaria indica* (Hausskn.) Pugsley**	Papaveraceae	A	0	5.64	0	0	0	Native	April-June	Therophyte
***Gagea lutea* (L.) Ker Gawl.**	Liliaceae	P	0	0	0	3.37	0	Native	April–June	Geophyte
***Galinsoga parviflora* Cav.**	Asteraceae	A	0	0	0	3.75	0	Exotic	June–Aug.	Therophyte
***Geranium rotundifolium* L.**	Geraniaceae	P	3.29	0	3.56	10.51	0	Native	June–July	Therophyte
***Iris kashmiriana* Baker**	Iridaceae	P	0	6.39	0	0	0	Native	April–Jun	Geophyte
***Lactuca serriola* L.**	Asteraceae	A	0	0	0	4.62	5.63	Native	July–Sept.	Therophyte
***Mazus pumilus (Burm.f.) Steenis***	Mazaceae	A	6.67	0	0	0	0	Native	April–May	Therophyte
***Medicago polymorpha* L.**	Fabaceae	A	0	0	7.92	9.81	0	Exotic	April–June	Therophyte
***Mentha arvensis* L.**	Lamiaceae	P	0	0	0	5.35	0	Native	July–Oct.	Hemicryptophyte
***Mentha longifolia* (L.) Huds.**	Lamiaceae	P	7.47	0	0	5.92	0	Exotic	July–Sept.	Hemicryptophyte
***Myosotis arvensis* (L.) Hill**	Boraginaceae	P	0	18.93	0	0	7.38	Exotic	May–June	Therophyte
***Nepeta cataria* L.**	Lamiaceae	P	0	0	0	3.37	0	Native	June–Aug.	Hemicryptophyte
***Oenothera rosea* L’Hér. ex Aiton**	Onagraceae	P	0	0	0	6.35	0	Native	April–Aug.	Therophyte
***Persicaria hydropiper* Delarbre**	Polygonaceae	A	56.71	14.92	3.91	9.36	6.05	Native	July–Sept.	Therophyte
***Phleum pratense* L.**	Poaceae	P	0	0	0	0	25.15	Native	July–Aug.	Hemicryptophyte
***Plantago lanceolata* L.**	Plantaginaceae	A-B-P	7.47	0	7.92	5.05	9.38	Native	May–Aug.	Therophyte
***Plantago major* L.**	Plantaginaceae	P	6.31	5.07	6.92	7.61	6.51	Native	May–Sept.	Hemicryptophyte
***Poa annua* L.**	Poaceae	A	95.64	42.03	99.22	34.75	21.26	Native	March–Aug.	Therophyte
***Poa bulbosa* L.**	Poaceae	P	0	0	0	10.54	0	Native	March–July	Hemicryptophyte
***Polygonum aviculare* L.**	Polygonaceae	A	4.96	18.55	5.28	9.39	10.27	Native	June–Aug.	Therophyte
***Polypogon fugax* Nees ex Steud.**	Poaceae	P	0	19.19	0	0	0	Native	May–June	Therophyte
***Ranunculus arvensis* L.**	Ranunculaceae	P	0	16.21	0	2.77	0	Native	March–April	Hemicryptophyte
***Ranunculus muricatus* L.**	Ranunculaceae	A-B-P	0	0	0	4.62	10.14	Exotic	April–June	Therophyte
***Ranunculus sceleratus* L.**	Ranunculaceae	P	15.53	5.91	16.13	0	0	Exotic	May–July	Therophyte
***Rorippa palustris* (L.) Besser**	Brassicaceae	A-B	8.64	21.17	0	0	0	Native	June–Aug.	Geophyte
***Rorippa indica* (L.) Hiern.**	Brassicaceae	A-B	0	5.76	0	0	0	Native	April–June	Geophyte
***Rumex dentatus* L.**	Polygonaceae	P	13.87	4.71	15.12	2.75	10.28	Native	June–July	Hemicryptophyte
***Senecio vulgaris* L.**	Asteraceae	A-B	0	0	0	2.08	0	Exotic	April–Sept.	Therophyte
***Sisymbrium loeselii* L.**	Brassicaceae	A	11.01	0	11.84	0	0	Exotic	June–Aug.	Therophyte
***Sonchus oleraceus* L.**	Asteraceae	A	0	0	0	0	5.62	Exotic	May–July	Therophyte
***Sorghum halepense* (L.) Pers.**	Poaceae	P	0	8.61	0	0	0	Exotic	June–Sept.	Geophyte
***Stellaria media* (L.) Vill.**	Poaceae	A	0	0	58.93	0	0	Native	March–Oct.	Therophyte
***Taraxacum officinale* Weber**	Asteraceae	P	0	3.31	0	5.73	7.41	Native	March–Aug.	Hemicryptophyte
***Trifolium pratense* L.**	Fabaceae	P	9.49	8.41	10.07	19.63	21.28	Native	May–Aug.	Hemicryptophyte
***Trifolium repens* L.**	Fabaceae	P	11.21	5.86	12.57	33.19	10.14	Native	May–Sept.	Hemicryptophyte
***Urtica dioica* L.**	Urticaceae	P	0	0	0	4.96	0	Exotic	June–Aug.	Therophyte
***Veronica anagallis-aquatica* L.**	Plantaginaceae	P	0	8.12	0	0	0	Native	June–Sept.	Hemicryptophyte
***Veronica peregrina* L.**	Plantaginaceae	A	0	0	0	0	13.47	Exotic	May–Oct.	Therophyte
***Veronica persica* Poir.**	Plantaginaceae	A	0	0	0	6.64	14.24	Native	March–July	Therophyte
***Vicia sativa* L.**	Fabaceae	A	5.21	4.71	6.31	0	0	Exotic	May–June	Therophyte

**Table 2:** Presence of weeds in the agriculture and horticulture systems of Kupwara, Jammu and Kashmir, India presented as different indices.

Indices	Agriculture			Horticulture	
	Paddy Field	Maize Field	Mustard Field	Apple Garden	Vegetable Garden
**Species number**	20	24	18	37	27
**Dominance**	0.153	0.062	0.164	0.048	0.045
**Shannon**	2.412	2.955	2.307	3.325	3.197
**Simpson**	0.847	0.937	0.835	0.951	0.955
**Evenness**	0.557	0.801	0.558	0.751	0.905

## Data Availability

All obtained data are provided in the research article.

## Supplementary Materials

The following supporting information can be downloaded at: https://www.mdpi.com/article/10.3390/plants12061222/s1, Table S1: Database of importance value index (IVI) of recorded species from different cropping systems of western Himalayas.
